# Detecting and Tracking β-Amyloid Oligomeric Forms and Dynamics In Vitro by a High-Sensitivity Fluorescent-Based Assay

**DOI:** 10.1021/acschemneuro.4c00312

**Published:** 2024-11-29

**Authors:** Yanyan Zhao, Oleksandr Brener, Ewa Andrzejewska, Jiapeng Wei, CloudOuterMan Reiß, Ole Tietz, Tuomas P. J. Knowles, Franklin I. Aigbirhio

**Affiliations:** † Molecular Imaging Chemistry Laboratory, Wolfson Brain Imaging Centre, Department of Clinical Neurosciences, University of Cambridge, Cambridge CB2 0QQ, U.K.; ‡ Institut für Physikalische Biologie, Heinrich-Heine-Universität Düsseldorf, Düsseldorf 40225, Germany; § Centre for Misfolding Diseases, Department of Chemistry, University of Cambridge,, Cambridge CB2 1EZ, U.K.; ∥ Dementia Research Centre, Department of Biomedical Sciences, Macquarie University, Sydney, NSW 2109, Australia

**Keywords:** amyloid oligomers, fluorescent probes, oligomer dissociation kinetics, Alzheimer’s disease, medicinal chemistry, neurological agents

## Abstract

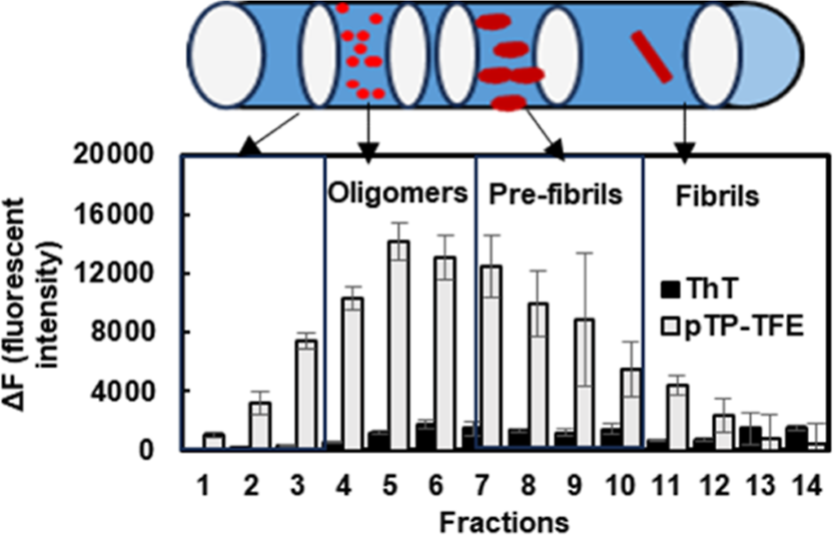

Aggregation of β-amyloid protein is a hallmark pathology of the neurodegenerative disorder Alzheimer’s disease and proceeds from monomers to insoluble misfolded fibril forms via soluble and highly toxic oligomeric intermediates. Given the dual feature of being the most toxic form of the Aβ aggregate proteome and an early marker of pathogenesis, there is a need for sensitive methods that can be used to detect Aβ oligomers and investigate the dynamics of aggregation. Herein, we describe a method based on the application of an oligomer-sensitive fluorescent chemical probe pTP-TFE combined with the use of a QIAD (Quantitative determination of Interference with Aβ Aggregate Size Distribution) assay to correctly identify Aβ oligomers in high sensitivity. pTP-TFE was evaluated and compared to thioflavin T and pFTAA, the two most widely used amyloid fibril dyes, and shown to be the only probe capable of detecting significant differences across all oligomeric species of β-amyloid. Furthermore, by observing changes in pTP-TFE fluorescence emission over time, we could track the dynamics of oligomer populations and thereby obtain kinetic information on the Aβ42 dynamic aggregation model. Therefore, we have established a highly sensitive, readily available, and simple method for studying β-amyloid protein aggregation dynamics.

## Introduction

**Figure 1:**
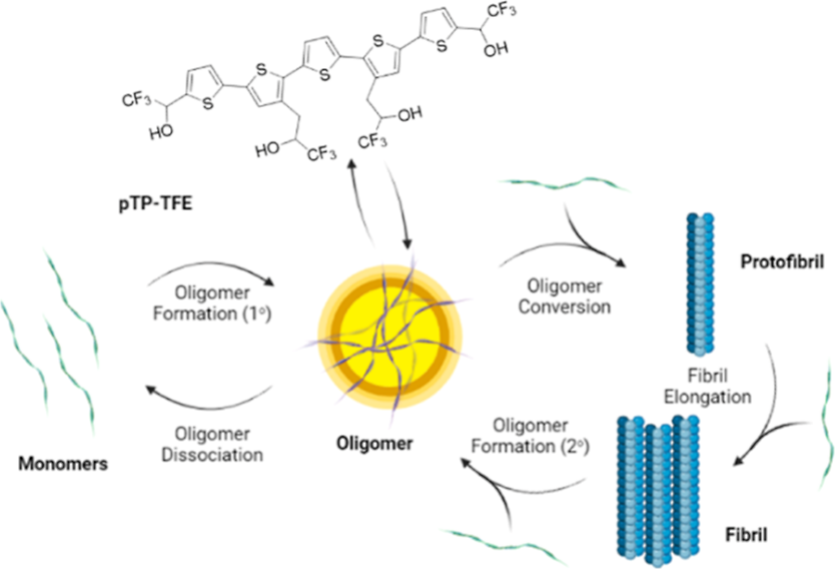
Fluorophore pTP-TFE binds to Aβ42 oligomers and monitors the dynamics of aggregation by detection primary oligomer formation (1°), oligomer dissociation, oligomer conversion, and secondary oligomer formation (2°).

Misfolded aggregates of β-amyloid (Aβ) protein are core pathology in Alzheimer’s disease (AD), where they are present as insoluble plaques in a number of brain regions.^1^ Other neurodegenerative diseases such as Parkinson’s disease, Huntington’s disease, and amyotrophic lateral sclerosis are also associated with misfolding and subsequent aggregation of Aβ protein.^2^ Monomeric Aβ42 and other length Aβ proteins self-assemble into fibrillar aggregates, which eventually manifest as plaques in patients suffering from advanced stages of the disease.^3^ The formation of insoluble Aβ fibrils proceeds via an intermediate state of low molecular weight aggregates, termed oligomers, which are soluble and are considered highly cytotoxic, as demonstrated in cellular and animal models.^4−7^ Recent studies have untangled the complex kinetics of Aβ42 fibrillization to demonstrate that all mature Aβ fibrils must originate as oligomers and that most Aβ42 oligomers dissociate into their monomeric precursors without forming new fibrils.^8^ These mechanistic insights suggest new and specific modes of therapeutic intervention to inhibit the formation of Aβ fibril formation. The minority of Aβ oligomers that convert into fibrillar structures are, therefore, important molecular targets in both the therapy and diagnosis of AD, given their dual function as the most toxic form of the Aβ aggregate proteome and as an early marker of pathogenesis. As a result, there is a need for sensitive methods that can be used to detect Aβ oligomers and investigate the dynamics of aggregation by temporal monitoring of oligomer populations. We recently developed a fluorescent pentameric oligothiophene (pTP), furnished with trifluoroethanol (TFE) functional groups, called pTP-TFE (Figure 1).^9^ Early results indicated a lag phase of pTP-TFE in comparison to the thioflavin T (ThT) kinetic curve in an Aβ aggregation assay, which suggests sensitivity for Aβ oligomers. These early results compared favorably to other work on oligomer sensitive fluorescence probes,^10^ BD-Oligo,^11^ AN-SP,^12,13^ and PTO-29.^14^

In this present study, we combine the use of pTP-TFE with the assay QIAD (Quantitative determination of Interference with Aβ Aggregate Size Distribution)^15^ which can purify different forms of Aβ42 presenting in the process of aggregation, from monomers to oligomers and fibrils, and quantify their concentration in solution to obtain pure and well-characterized Aβ42 oligomer analytes. We demonstrate that these oligomers can then be detected by using pTP-TFE and that monitoring fluorescence intensity over time can elucidate aggregation dynamics. The dynamics we obtain from this method correlate well with recently published findings using the more complex technique using tritium isotope-labeled recombinant Aβ42.^8^ Therefore, in contrast to the isotope-^8^ or NMR-based^16^ aggregation studies, which are costly, difficult to adapt, and require specialized equipment, the use of our fluorescent probe for detecting Aβ oligomers and studying aggregation dynamics provides a cheaper, readily available, and simpler method to use. Crucially, detection of Aβ42 aggregate species depends on direct emission of a high quantum yield fluorophore and therefore promises to be more sensitive than approaches that rely on relatively insensitive Förster resonance energy transfer (FRET) detection.^17^ Herein, we present our findings.

## Results and Discussion

### QIAD Assay

**Figure 2:**
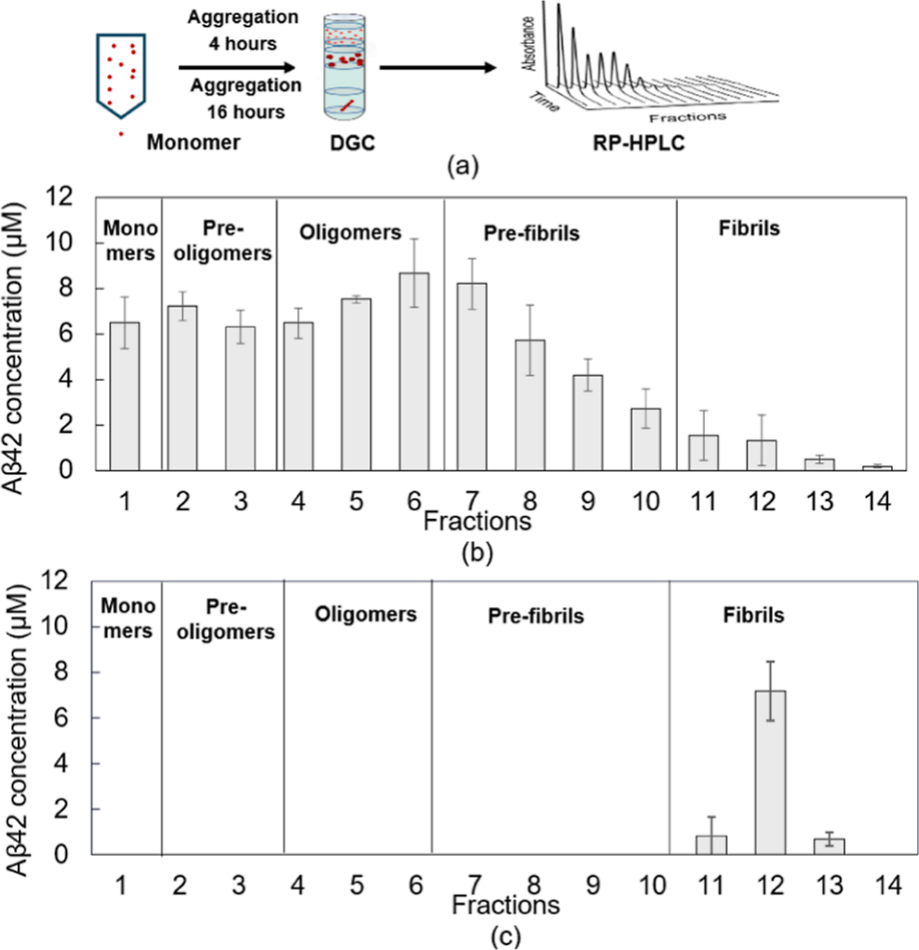
(a) QIAD workflow—monomers are left to aggregate at room temperature while shaking and are separated by density gradient ultracentrifugation (DGC), followed by characterization on RP-HPLC, to produce pure Aβ42 aggregates. (b,c) Concentration of Aβ aggregates in fractions obtained from DGC after incubation of Aβ42 monomers (80 μM) for 4 h (b) and Aβ42 monomers (10 μM) for 16 h (c) by RP-HPLC under harsh conditions (80 °C column temperature). *N* = 3 (mean ± SD).

We obtained Aβ42 oligomers by aggregation of a recombinant Aβ42 monomer, followed by purification and quantification using the QIAD assay^15^ (Figure 2a). QIAD uses DGC in a discontinuous gradient of iodixanol (layered in bands of 5% to 50% w/v) to separate Aβ aggregate species. This method is matrix-free and fractionates Aβ aggregates according to their sedimentation coefficients, which are dependent on the particle size and shape. Fractions were characterized using reverse phase high pressure liquid chromatography (RP-HPLC) under harsh conditions (80 °C column temperature) to affect the breakdown of aggregates into monomers, followed by integration of absorption signals to determine the total amount of Aβ42 in each fraction of aggregates. Total disassembly of aggregates allows quantitative analysis of the Aβ42 content by converting all Aβ assemblies into a single species with the same retention time.

Incubation of monomeric, recombinant Aβ42 for 4 h at room temperature while shaking followed by QIAD purification and analysis shows formation of oligomers, with few fibrils present (Figure 2b), while incubation for 16 h leads as expected to the formation of fibrils as the dominant species (Figure 2c). This purification method has been thoroughly validated^15^ and has demonstrated that fraction 1 contains Aβ monomers only; fractions 2 and 3 are classified as preoligomers, characterized by a molecular weight of less than 66 kDa; fractions 4–6 are identified as Aβ oligomers, based on circular dichroism measurements showing a β-sheet spectrum, however, with no ThT fluorescence observed;^15^ fractions 7–10 contain high molecular weight Aβ assemblies, termed prefibrils, while fractions 11–14 correspond to Aβ fibrils.^15^ It is important to note that adjacent fractions may overlap between groups; for instance, fraction 7 may be considered an oligomer, and fraction 11 may be grouped with prefibrils.

### Characterization of pTP-TFE Binding to Oligomeric Aβ

**Figure 3:**
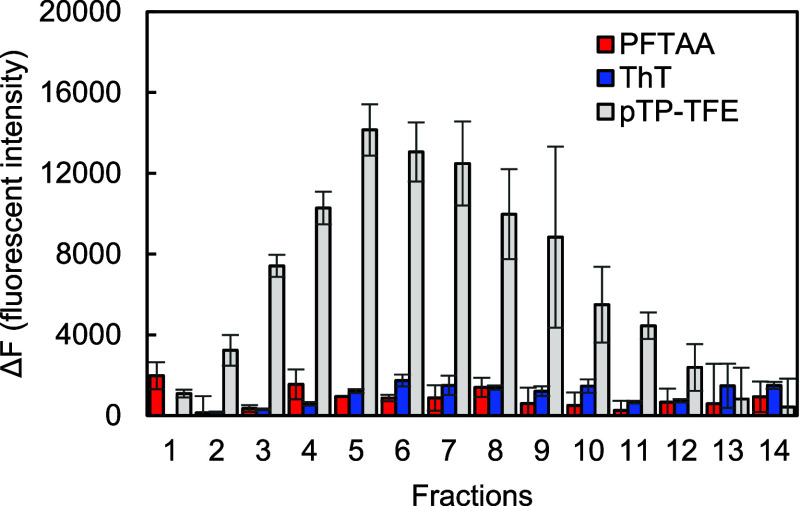
Fluorescence intensity changes of ThT, pFTAA, and pTP-TFE after interaction with Aβ42 fractions from DGC (Figure 2b). ThT (blue, 100 nM), pFTAA (red, 100 nM), pTP-TFE (gray, 100 nM) excited at 450 nm, emission at 520 nm. *N* = 3 (mean ± SD).

With well-defined Aβ42 oligomers in hand, we sought to establish whether the fluorescent probe pTP-TFE can specifically detect these aggregate species. To this end, we applied three fluorescent probes, pTP-TFE, ThT, and pFTAA,^18^ separately to freshly prepared and fractionated Aβ42 aggregates (4 h incubation) at protein concentrations determined by RP-HPLC (Figure 2b). Self-fluorescence of ThT, pFTAA, and pTP-TFE was determined and fluorescence signals due to interaction with iodixanol obtained from a density gradient run without Aβ (Figure S1). This control data was used to normalize fluorescent signals obtained with Aβ42 aggregates shown in Figure 3. pTP-TFE showed 15 times greater fluorescence intensity in the presence of Aβ oligomers (fraction 5) when compared to Aβ monomers (fraction 1). In comparison, the fluorescence intensity of ThT was only 2 times greater, while pFTAA slightly decreased in fluorescence when incubated with the same aggregate species. These results demonstrate that pTP-TFE can bind to preoligomer fractions 2 and 3, while maximum fluorescence intensity change is observed with Aβ oligomers (fraction 5), and no fluorescence increase with fraction 1 was detected. This renders pTP-TFE a far more powerful tool for the study of Aβ aggregation than ThT or pFTAA, which show little to no interaction with oligomeric Aβ.

**Figure 4:**
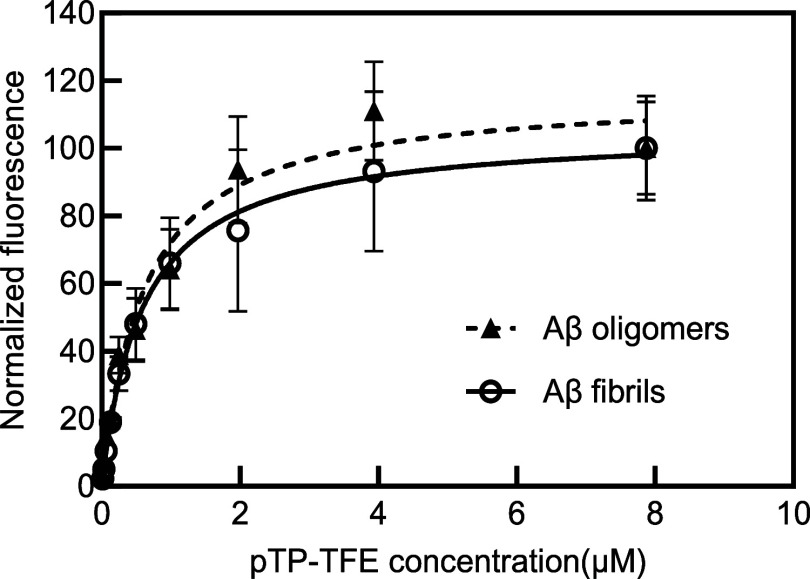
Binding affinity dose–response curve of pTP-TFE with Aβ oligomers (fractions 5 with a concentration of 1.47 μM) and fibrils (fraction 12 with a concentration of 1.59 μM). One specific site binding model is used here to obtain the *K*_d_. Excitation at 450 nm, emission at 520 nm. *N* = 3 (mean ± SD).

The fluorescence intensity of pTP-TFE is lower when bound to Aβ prefibrils in comparison to oligomers, which might be attributable to protein concentration difference between fractions. As shown in Figure 2b, the Aβ concentration declines in fractions 8–14. Indeed, pTP-TFE bound to Aβ fibrils obtained by incubating Aβ42 monomers for 16 h, resulting in protein samples of comparable concentration (Figure 2c, fraction 12) to Aβ oligomers (Figure 2b, fraction 5), shows similar fluorescence intensity when compared to Aβ oligomer binding (Figure S2). Full-spectrum scans of both ThT and pTP-TFE with Aβ42 incubated for 4 and 16 h were performed and are presented in Figure S3. The results show that pTP-TFE produces similar intensity levels for both Aβ42 samples, whereas ThT exhibits a 10-fold increase in intensity with fibrils. The binding affinities of pTP-TFE with Aβ oligomers (fraction 5) and Aβ fibrils (fraction 12) are shown in Figure 4. This result indicates comparable affinities, with the interaction between pTP-TFE and Aβ oligomers (fraction 5) having a fitted *K*_d_ value of 0.61 ± 0.11 μM, while the binding affinity with Aβ fibrils (fraction 12) is fitted at 0.59 ± 0.12 μM.

**Figure 5:**
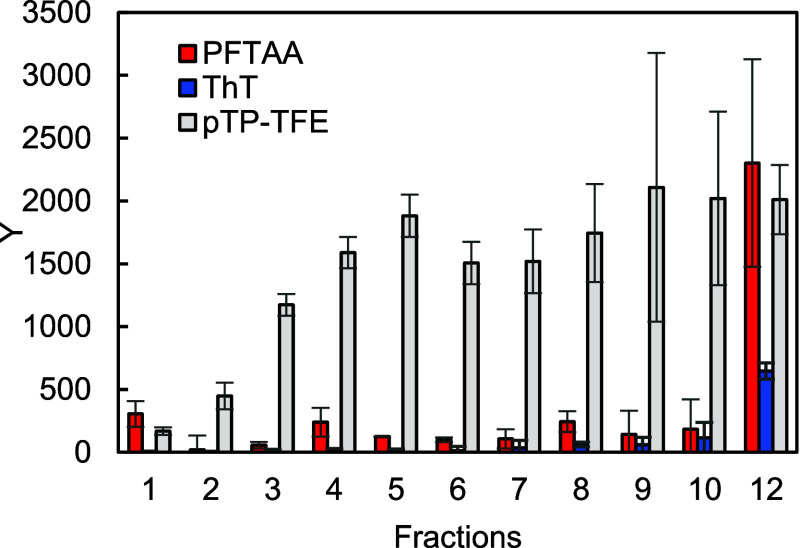
Fluorescence intensity normalized for protein concentration using eqs 1 and 2. Fractions 1–10 with Aβ42 aggregates obtained from 4 h incubation, and fraction 12 with aggregates from 16 h incubation. *N* = 3 (mean ± SD).

To rule out that the differences in fluorescence intensity observed in Figure 3 are due to fluctuations in protein concentration, we next normalized our findings to quantitative measures of the Aβ42 protein content. To this end, we introduced a fluorescence intensity per unit of protein value “Y” to evaluate the ability of all three compounds to bind a specific Aβ42 species. Due to the low amount of fibrils (fractions 11–14) obtained from 4 h incubation experiments, we used fibrils obtained from 16 h incubation of Aβ42 (Figure 2c, fraction 12) to evaluate and normalize the ability of compounds to bind Aβ fibrils. Calculation of fluorescence intensity value “Y” is shown in eqs 1 and 2, and the results are presented in Figure 5.
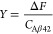
1
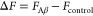
2

As shown in Figure 5, both ThT and pFTAA have the highest fluorescence intensity with Aβ42 fibrils (fraction 12) and no association with oligomeric forms of Aβ42. This is in good agreement with published literature and reiterates that both ThT and pFTAA are Aβ fibril fluorescent probes.^18,19^ In contrast, pTP-TFE shows significantly higher fluorescence intensity than ThT and pFTAA with all oligomeric species, indicating its potential as a probe for detecting Aβ oligomer forms and tracking the dissociation of oligomers and prefibrils.

### Identification of Aβ Oligomers by pTP-TFE/ThT Ratio

**Figure 6:**
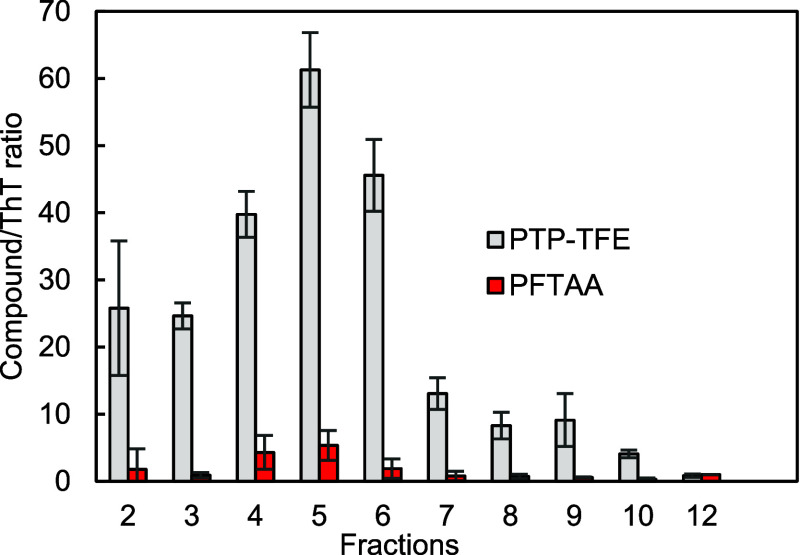
Normalized fluorescence ratios of compounds (pTP-TFE and pFTAA) and ThT with Aβ preoligomers and oligomer fractions (2–10) and fibril fraction 12. The concentrations of all compounds are at 100 nM. Excitation at 450 nm, emission at 520 nm. *N* = 3 (mean ± SD).

While pTP-TFE shows good affinity for Aβ oligomers, it also binds to Aβ fibrils. To overcome this limitation, we next investigated the pTP-TFE/ThT ratio (pTR). To this end, pure fractions of oligomeric and fibril Aβ species (Figure 2) were treated with pTP-TFE and ThT separately, and fluorescence intensities were recorded and adjusted for protein content (eqs 1 and 2). Results were then normalized for maximum fluorescence, observed with Aβ fraction 9 for pTP-TFE and Aβ fibrils fraction 12 for ThT. The ratio of pTP-TFE over ThT normalized fluorescence accurately detects the presence of Aβ oligomers in comparison to preoligomers and fibrils (Figure 6). Preoligomers fractions give pTR values of approximately 20, while Aβ oligomers fractions give ratios in excess of 30 and as high as 60, making oligomeric species clearly identifiable using pTR.From oligomers to prefibrils, pTR values rapidly decrease approximately to 10, clearly demonstrating the selectivity of this assay. pTR values for Aβ fibrils are close to one, resulting in the selectivity of Aβ oligomers over Aβ fibrils in excess of 30 for pTR.

By comparison, a pFTAA–ThT ratio, conducted using the same methodology, showed only modest selectively for oligomers (Figure 6). pFTAA to ThT ratios for Aβ oligomers were found to be <5, while ratios for Aβ fibrils remained at approximately one. Indeed, a direct comparison of pTR and pFTAA to ThT ratios (Figure 6) demonstrates a significant (13.2 ± 6.25) difference across all oligomeric species of Aβ. The ratio of pTP-TFE to ThT is an order of magnitude greater than for pFTAA, highlighting the utility of pTR in specifically identifying oligomeric species of Aβ. The discovery of an oligomer-sensitive pharmacophore is essential for the development of screening tools that will aid the detection of Aβ oligomers in biological samples. The approach pioneered in this work uses a pTP-TFE/ThT ratio to distinguish Aβ oligomers from Aβ fibrils. Aβ oligomers were detected at a pTP-TFE/ThT ratio in excess of 60, while ratios drop dramatically with Aβ prefibrils (<10) and Aβ fibrils (=1). pTR is cheap and convenient while retaining or exceeding the sensitivity of methods using isotope labeling, NMR, or FRET.^8,16,17^ In addition, these results point toward a particular sensitivity of pTP-TFE to conformational changes from Aβ monomers to oligomers, suggesting that pTP-TFE can be used to probe the molecular mechanism of Aβ42 aggregation.

### Dissociation of Aβ Oligomers

**Figure 7:**
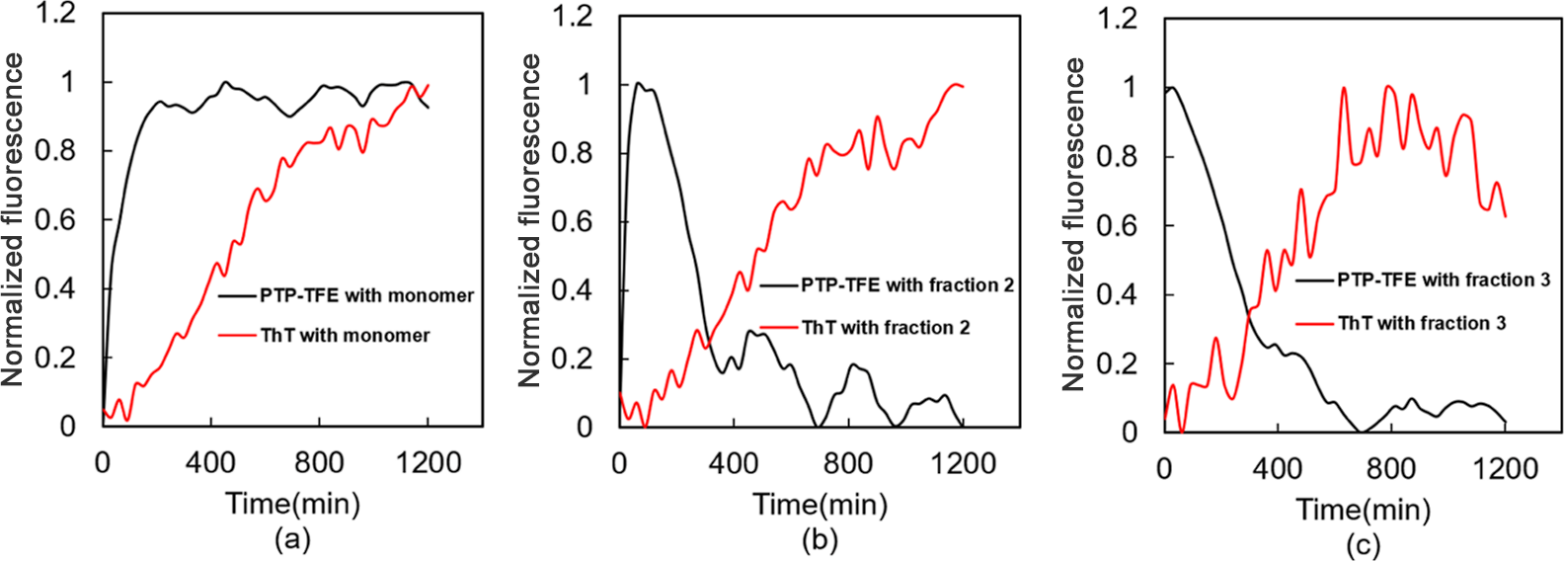
Aggregation kinetics of (a) monomer fraction 1, (b) preoligomer fraction 2, and (c) preoligomer fraction 3, probed with pTP-TFE and ThT for 1200 min under aggregating conditions (25 °C, no shaking). Excitation at 450 nm, emission at 520 nm (*N* = 3).

To determine whether dynamics of Aβ42 aggregation can be studied using pTP-TFE, we used isolated monomers, preoligomers, oligomers, prefibrils, and fibrils (fractions 1 to 14, Figure 2b) and monitored pTP-TFE binding by fluorescence readout over a time of 20 h at 25 °C without shaking. ThT displayed similar kinetic profiles for monomer fraction 1 and preoligomer fractions 2 and 3 (Figure 7a–c). This indicates that ThT is not sensitive to preoligomeric Aβ42. pTP-TFE, on the other hand, showed a lag-free biphasic assembly kinetic with Aβ42 monomers (Figure 7a) and exhibited distinct and significant differences between the monomer fractions and the preoligomeric fractions (Figure 7b,c). This disparity in response suggests that pTP-TFE possesses sensitivity toward oligomeric Aβ42 and can provide insights into the dissociation process of these oligomers.

**Figure 8:**
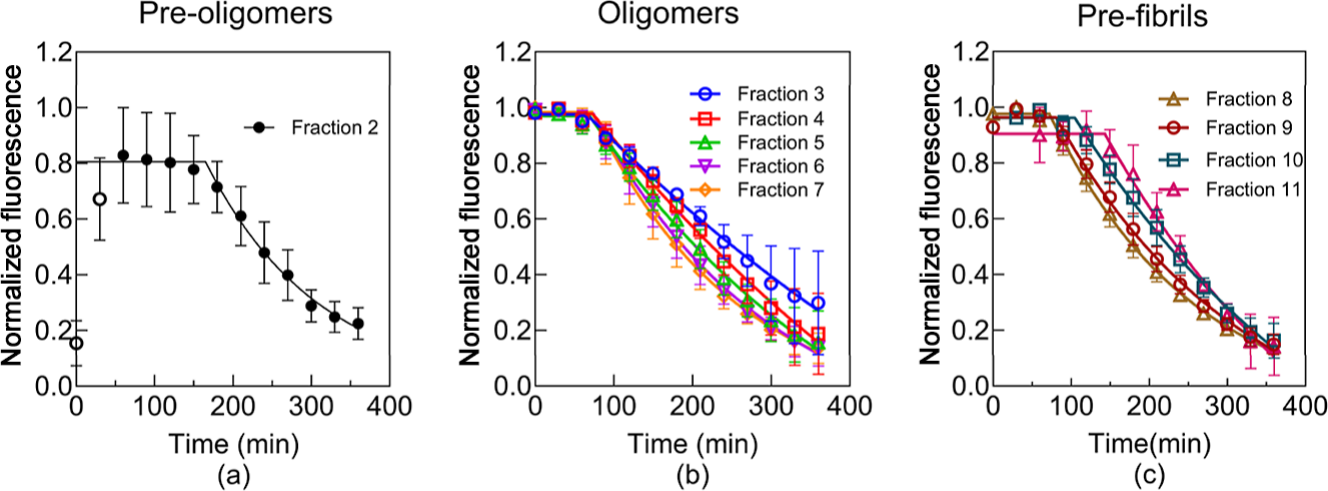
Aβ42 dissociation kinetics of preoligomer fraction 2 (a), oligomers fractions 3–7 (b), and prefibril fractions 8–11 (c) probed with pTP-TFE for 360 min under aggregating conditions (25 °C, no shaking) fitted with a plateau followed one-phase decay model by GraphPad Prism. (a) Preoligomer fraction 2 with pTP-TFE fluorescence. The dissociation constant is 1.1 × 10^–4^ s^–1^. Fraction 2 exhibits the highest instability among the oligomers, primarily because it is the fraction closest to monomers. (b) Oligomer fractions 3–7 with pTP-TFE fluorescence. The dissociation constants for fractions 3–7 are increased as the oligomer size increases. Dissociation constants are K_3_ = 2.0 × 10^–5^ s^–1^, K_4_ = 2.4 × 10^–5^ s^–1^, K_5_ = 4.3 × 10^–5^ s^–1^, K_6_ = 7.3 × 10^–5^ s^–1^, and K_7_ = 9.0 × 10^–5^ s^–1^. (c) Prefibril fractions 8–11 with pTP-TFE fluorescence. The dissociation constants begin to decline starting at fraction 8, coinciding with the oligomer size approaching that of fibrils. Dissociation constants are K_8_ = 8.9 × 10^–5^ s^–1^, K_9_ = 6.9 × 10^–5^ s^–1^, K_10_ = 4.5 × 10^–5^ s^–1^, and K_11_ = 4.9 × 10^–5^ s^–1^. *N* = 3 (mean ± SD).

Notably, fractions 4 to 9 exhibited intriguing similarities in the fluorescence signals observed with both ThT and pTP-TFE (Figure S4). These signals followed a consistent pattern characterized by two distinct stages of decrease at 360 and 720 min. The one-phase decay model was employed to fit all the data points illustrated in Figure S5. However, the representation of the fitting results was not satisfactory due to the variation in the data over long times (Figure S5). As a result, the data points ranging from 360 to 1200 min were excluded for fitting using the one-phase decay model. The resulting outcomes are depicted in Figure 8. The smallest fraction, preoligomer fraction 2, exhibits a rapid dissociation rate of 1.1 × 10^–4^ s^–1^ (Figure 8a). This value aligns with the previously reported oligomer dissociation rate (1.0 × 10^–4^ s^–1^) determined using sensitive isotopic labeling techniques.^8^ Moving from fraction 3 to fraction 7, an increase in the oligomer size corresponds to an increase in the dissociation rate (Figure 8b). This observation suggests that larger oligomers in fractions 6 and 7 are less stable than oligomers at fractions 4 and 5. However, once the oligomers undergo conversion to prefibrils, the dissociation rate decreases (Figure 8c). This is attributed to the fact that the prefibrils’ structure is more stable compared to oligomers. Additionally, we observed that the dissociation rates of fractions 7 and 8 are approximately 9.0 × 10^–5^ s^–1^. This value aligns with the preoligomer dissociation rate 1.1 × 10^–4^ s^–1^ (Figure 8a). The preoligomer signal (Figure 8a) increases in the beginning and then starts dissociation, which indicates preoligomers first converted to oligomers and prefibrils before beginning to dissociate. This observation provides a direct proof of pTP-TFE’s utility as a sensitive and facile tool to study the dissociation kinetics of Aβ42 oligomers. Figure 8 also shows that all oligomer fractions have stable fluorescence with pTP-TFE during the initial 60 min period. This observation indicates that the oligomers might associate to big size aggregates like prefibrils. This does not change the fluorescence intensity of pTP-TFE as indicated in Figure 5. Once a small amount prefibrils are formed, it can surface-catalyze oligomer dissociation and the fluorescent signal of pTP-TFE decreases quickly.^20^ Furthermore, pTP-TFE can serve as a valuable fluorophore reference for assessing the stability of the Aβ42 oligomers.

For the Aβ42 fibrils (fractions 12–14, Figure 2b), fluorescence signals detected with both ThT and pTP-TFE are presented in Figure S6. Given that the Aβ42 concentration in fraction 13 is below 0.62 μM and in fraction 14 is below 0.25 μM, the observed signals for both ThT and pTP-TFE in Figure S6 (b,c,d) primarily reflect the gradient buffer solution itself. In fraction 12, only one group has an Aβ42 concentration of 2.6 μM Figure S6(a), while the other two groups have concentrations below 0.68 μM. Consequently, we observed fluorescence signals in just one group of Aβ42 fibrils, where the pTP-TFE signal increased from 4557 to 7017, in contrast to the consistently decreasing signals for Aβ42 oligomers. This suggests that fibrils might dissociate into prefibrils, but further investigation is needed in future studies to confirm this.

It is interesting that the dissociation rate increases from fraction 3 to 7 and then decreases, suggesting that the larger aggregates of a certain structure are less stable and then convert to prefibrils. It is evidence of different structures being present. Another significant observation we report is that pTP-TFE demonstrates fluorescence sensitivity toward Aβ42 preoligomers. In contrast, ThT does not exhibit such sensitivity toward these smaller oligomeric forms. This finding highlights the unique capability of pTP-TFE to detect and respond to oligomeric Aβ42 species with a limited number of monomer units. The most recently reported dynamic model of Aβ42 oligomers suggests that most oligomers dissociate to monomers with the rate at 9 × 10^–5^ s^–1^.^8^ The profiles of oligomer fractions 7 and 8 obtained using pTP-TFE are in good agreement with this finding. pTP-TFE captures these characteristics well and can be used as a fluorescent probe to measure the Aβ42 oligomer dynamics and assess the stability of Aβ42 oligomers, with potential utility in drug discovery.

## Conclusions

In this work, we demonstrate that pTP-TFE is a highly sensitive fluorophore for detecting oligomers of Aβ. Combined with a QIAD assay, we applied pTP-TFE to correctly identify Aβ oligomers using a simple pTP-TFE/ThT ratio value. pTP-TFE was evaluated and compared to ThT and pFTAA, the two most widely used amyloid fibril dyes, and shown to be the only probe capable of detecting significant differences across all oligomeric species of β-amyloid. Furthermore, with our method, we could obtain kinetic information on the Aβ42 dynamic aggregation model by observing changes in fluorescence emission over time. Therefore, we established a readily available and simple method to use for studying aggregation dynamics. This technique we foresee could provide a powerful platform to aid the development of sensitive assays to screen novel therapeutics designed to combat protein misfolding diseases at specific stages of the aggregation pathway.

## Methods

### Synthesis of pTP-TFE

The synthesis of pTP-TFE followed a published procedure.^9^ Briefly, the synthesis of pTP-TFE relied on the trifluoromethylation of individual thiophene building blocks and their subsequent assembly to the pentameric target compound through Suzuki cross-coupling reactions. pTP-TFE was stored as a lyophilized powder in dark conditions in a −20 °C freezer prior to usage.

### Preparation of Aβ42 Peptide Solutions

To dissolve any pre-existing aggregates of Aβ42 and to ensure the monomeric state of Aβ42, the sample was predissolved in 1,1,1,3,3,3-hexafluoro-2-propanol (HFIP; Sigma-Aldrich, Germany) at 1.43 mg/mL and incubated overnight at room temperature. HFIP was removed by evaporation for 30 min in the hood, followed by a drying step in a centrifugal evaporator (RVC 2–18, Christ, Mainz, Germany) for a further 30 min. The dried Aβ42 was stored at −20 °C until use.

## QIAD Assay

The QIAD procedure followed published protocols.^13^ Briefly, 80 μM Aβ42 monomers were incubated 4 h and 10 μM Aβ42 monomers were incubated16 h for the oligomer and fibril assay respectively. Both incubations were done in 10 mM sodium phosphate buffer (pH 7.4) at RT and shaking (600 rpm). A discontinuous gradient of iodixanol was preformed by layering 260 μL of 50% (w/v) iodixanol at the bottom of an 11 × 34 mm polyallomer centrifuge tube, overlaid by 260 μL of 40% (w/v), 260 μL of 30% (w/v), 780 μL of 20% (w/v), 260 μL of 10% (w/v), and 100 μL of 5% (w/v) iodixanol. The total volume of the nonlinear gradient, buffered with 10 mM sodium phosphate (pH 7.4) and including 100 μL Aβ42 sample on top, was 2020 μL. The samples were spun at 259,000*g* for 3 h at 4 °C in a TL 100 ultracentrifuge with a TLS-55 rotor (both Beckman Instruments, Brea, USA). After the centrifugation, 14 fractions of 140 μL were harvested with a pipet by upward displacement.

All of the fractions were analyzed with respect to their Aβ42 content by analytical RP-HPLC. Quantifications of Aβ42 present in DGC fractions were performed by RP-HPLC on a Zorbax SB-300C8 column (5 μ m, 4.8 × 250 mm, Agilent, Waldbronn, Germany) connected to an Agilent 1260 Infinity system.

### Fluorescence Test of Aβ42 Fractions with a Plate Reader

pTP-TFE, pFTAA, and ThT fluorescence were measured in freshly prepared Aβ42 fractions (1–14) and in fractions from a density gradient run without Aβ42 as an iodixanol control after addition of these three compounds separately. The fluorescent intensities are measured in 96-well plates (Nunclon 96 Flat Bottom) with a lid, black, μclear at room temperature 25 °C. A Tecan M1000pro plate reader (Institute of Physical Biology, Heinrich-Heine-University of Düsseldorf) was used to read out the fluorescence intensity with excitation wavelength 450 nm, emission wavelength 520 nm, excitation bandwidth 5 nm, emission bandwidth 5 nm, gain 150 manual, number of flashes 10, and flash frequency 400 Hz. To determine the ratios of pTP-TFE and pFTAA relative to ThT, the fluorescence intensities were first adjusted by subtracting the values from monomer fraction 1 and then normalized to the maximum fluorescence observed: Aβ fraction 9 for pTP-TFE and Aβ fibril fraction 12 for ThT and pFTAA. Since the fluorescence intensity in fraction 1 is zero for all three compounds, it was excluded from the ratio calculation.

### Binding Affinity of pTE-TFE with Aβ42 Oligomers and Fibrils

pTP-TFE 20 μM DMSO was used as a stock solution. A series of dilutions of pTP-TFE (50 μL) from 10 to 0.02 μM were prepared to buffer NaPi for the binding affinity test with Aβ42 oligomers (fraction 5) and to PBS buffer for the test of Aβ42 fibrils (fraction 12). 13.5 μL of Aβ42 from fraction 5 (6.91 μM) and fraction 12 (7.51 μM) was separately added to each pTP-TFE dilutions with three groups of repeats at each concentration point. Control experiments were done by adding 13.5 μL of buffer to each pTP-TFE dilutions. The preparation was transferred to the 96-well plates with a lid, black μclear. A Tecan S2 plate reader (Institute of Physical Biology, Heinrich-Heine-University of Düsseldorf) was used to read out the fluorescence intensity with excitation wavelength 450 nm, emission wavelength 520 nm, excitation bandwidth 5 nm, emission bandwidth 5 nm, gain 150 manual, number of flashes 10, and flash frequency 400 Hz.

The data were calculated by the fluorescence intensity from pTP-TFE with Aβ42 fraction 5/12, reducing the value of pTP-TFE with buffer. The binding affinities were fitted by nonlinear fitting of one specific binding model with the software GraphPad Prism.

### pTP-TFE Profiling of Aβ42 Oligomer Dissociation

Fractions 1–8, obtained from the QIAD assay after a 4 h incubation of Aβ42, were each supplemented with 0.1 μM pTP-TFE. A Tecan M200 plate reader (Institute of Physical Biology, Heinrich-Heine-University of Düsseldorf) was used to obtain the kinetic curve of all the fraction aggregation. 40 cycles of fluorescence intensity readout were set up with each cycle 30 min, excitation wavelength 450 nm, and emission wavelengths from 480 to 520 nm. All fractions were incubated at 25 °C without shaking. All data are fitted with a plateau followed by one-phase decay model by GraphPad Prism.

## Supporting Information Available

Fluorescence data, excitation and emission spectrums, data fitting, and Aβ42 concentrations mesured by HPLC. (PDF)

## Abbreviations

Aβ: amyloid beta
Aβ42: amyloid beta (1–42)
AD: Alzheimer’s disease
pTP: pentameric oligothiophene
TFE: trifluoroethanol
ThT: thioflavin T
QIAD: Quantitative determination of Interference with Aβ Aggregate Size Distribution
DGC: density gradient ultracentrifugation
RP-HPLC: reverse-phase high-performance liquid chromatography
pTR: pTP-TFE–ThT ratio
FRET: Förster resonance energy transfer
HFIP: 1,1,1,3,3,3-hexafluoro-2-propanol
