# Supplementary material for: Detecting and Tracking β-Amyloid Oligomeric Forms and Dynamics In Vitro by a High-Sensitivity Fluorescent-Based Assay
Source: ACS Chem Neurosci. 2024 Nov 29;15(24):4383–9. doi: 10.1021/acschemneuro.4c00312 (PMC11660153; doi:10.1021/acschemneuro.4c00312)
Supplement: Supplementary file 1 — cn4c00312_si_001.pdf [file cn4c00312_si_001.pdf]

Supplementary Information

Detecting and tracking β-amyloid oligomeric forms and dynamics in vitro by a high sensitivity fluorescent based assay

Yanyan Zhao, [†1] Oleksandr Brener, [†2] Ewa Andrzejewska,[3] Jiapeng Wei,[3] CloudOuterMan Reiß, [2] Ole Tietz,[4] Tuomas Knowles,[3] and Franklin Aigbirhio[*1]

1. Molecular Imaging Chemistry Laboratory, Wolfson Brain Imaging Centre, Department of Clinical Neurosciences, University of Cambridge, CB2 0QQ, Cambridge, United Kingdom.
2. Institut für Physikalische Biologie, Heinrich-Heine-Universität Düsseldorf, 40225, Düsseldorf, Germany
3. Centre for Misfolding Diseases, Department of Chemistry, University of Cambridge, CB2 1EZ, Cambridge, UK
4. Dementia Research Centre, Department of Biomedical Sciences, Macquarie University, Sydney, NSW, 2109, Australia

† These authors contributed equally to this work.

* Corresponding author E-mail: fia20@medschl.cam.ac.uk.

Table of contents:

1. Fluorescence data for ThT, pFTAA and pTP-TFE in Iodixanol fractions S3
2. PTP-TFE raw fluorescence data in Aβ fractions S4
3. Excitation and emission spectrums for ThT and pTP-TFE S5
4. Kinetic fluorescence data of ThT and pTP-TFE for fractions 4-11 S6
5. Data fitting with a one-phase decay model S7
6. Kinetic fluorescence data for fibril-specific fractions S8
7. Aβ $_{(1-42)}$ concentrations by HPLC S9

Supporting figures

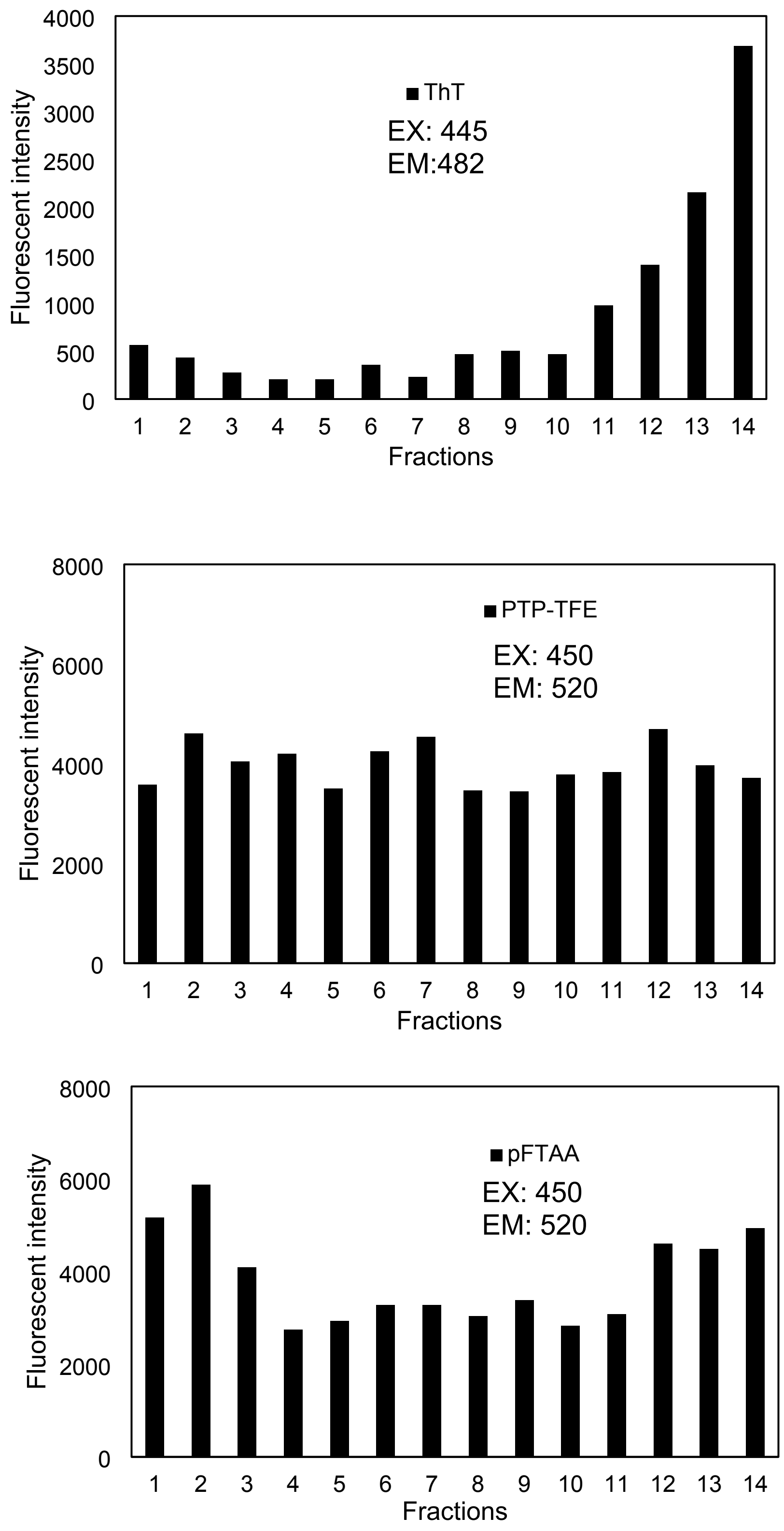

Figure S1: ThT (100nM), pTP-TFE (100nM) and pFTAA (100nM) fluorescent intensity controls in Iodixanol fractions 1-14.

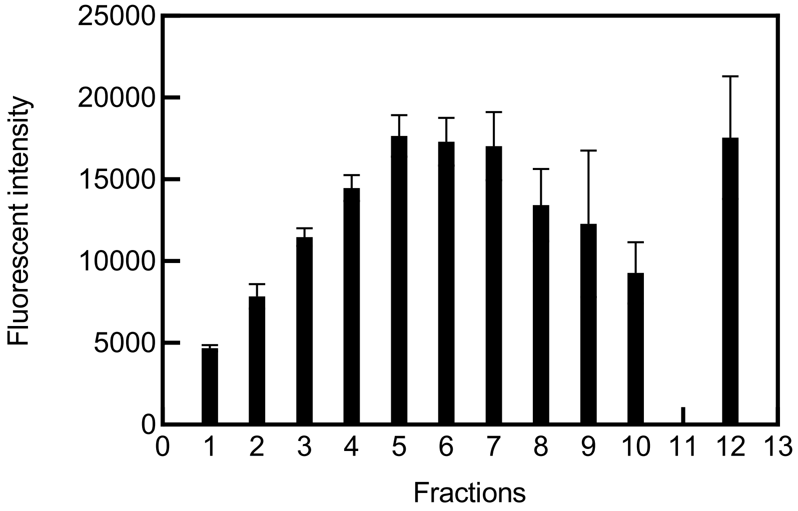

Figure S2: PTP-TFE fluorescent intensity with Aβ42 monomers, pre-oligomers, oligomers, pre-fibrils and fibrils.

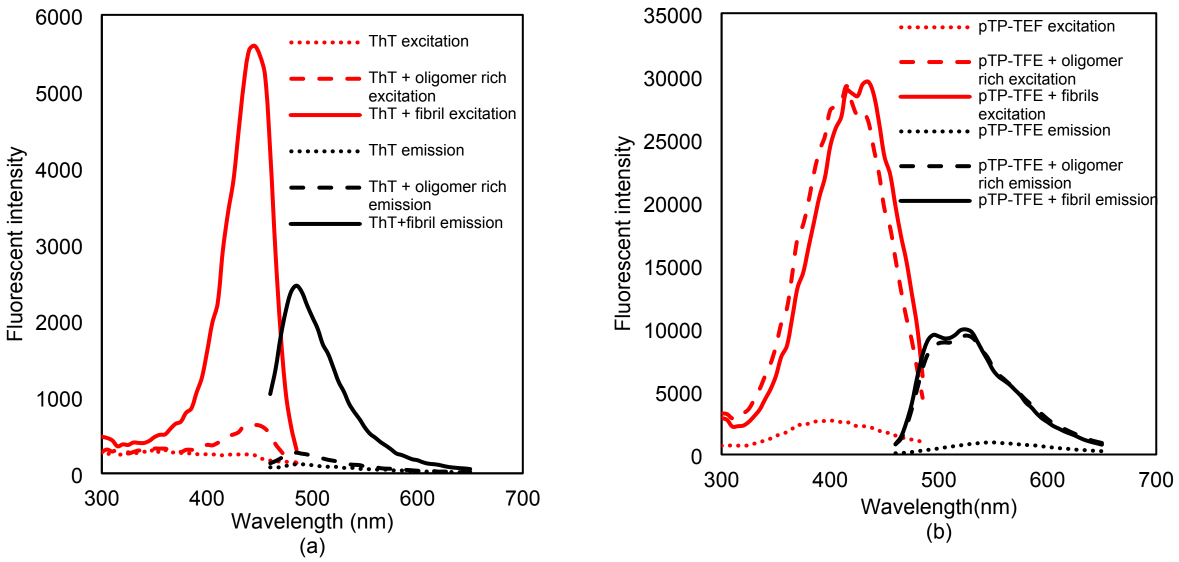

Figure S3: (a) ThT (5 μM) and (b) pTP-TFE (5 μM) excitation and emission spectrum scan with Aβ42 oligomers (8 μM), fibrils (8 μM) and PBS buffer. Aβ42 monomer incubated at room temperature for 4 hours to generate the oligomer rich Aβ42 and 16 hours to generate the fibrils.

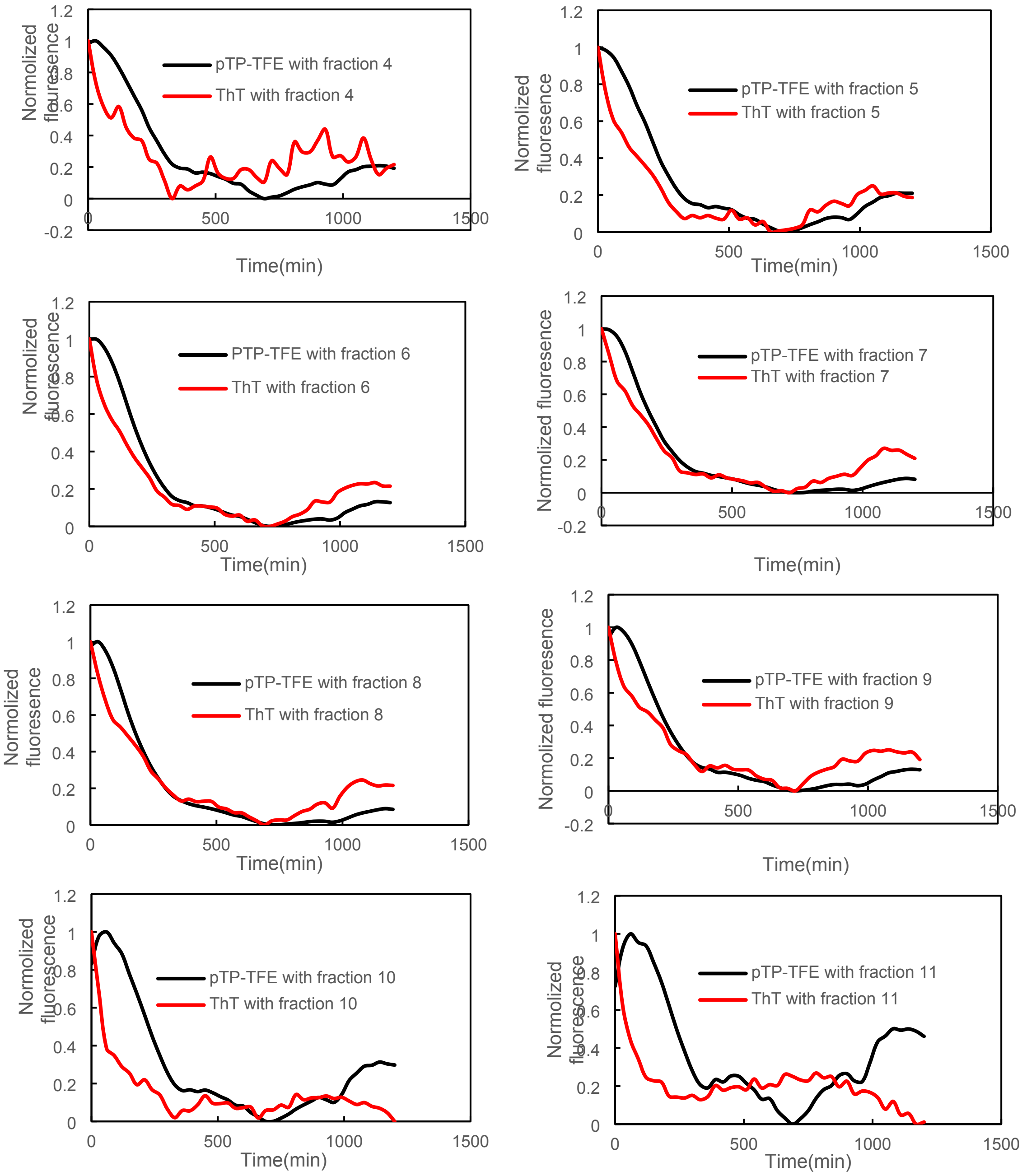

Figure S4: Kinetics of Aβ42 fractions (4-11) with pTP-TFE and ThT normalized fluorescence.

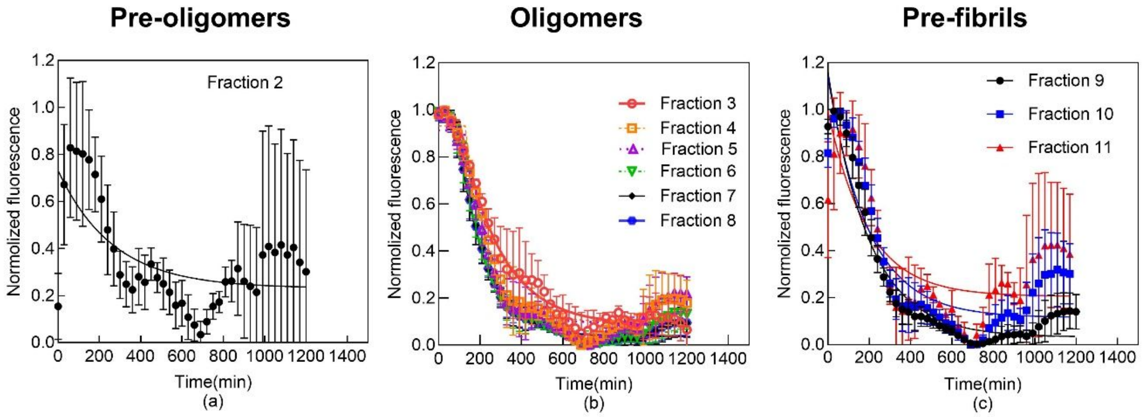

Figure S5: All data are fitted with plateau value followed one phase decay using GraphPad Prism. (a) pre-oligomer fraction 2 with pTP-TFE fluorescence. (b) Oligomer fraction 3-7 with pTP-TFE fluorescence. (c) pre-fibril fractions 8-11 with pTP-TFE fluorescence.

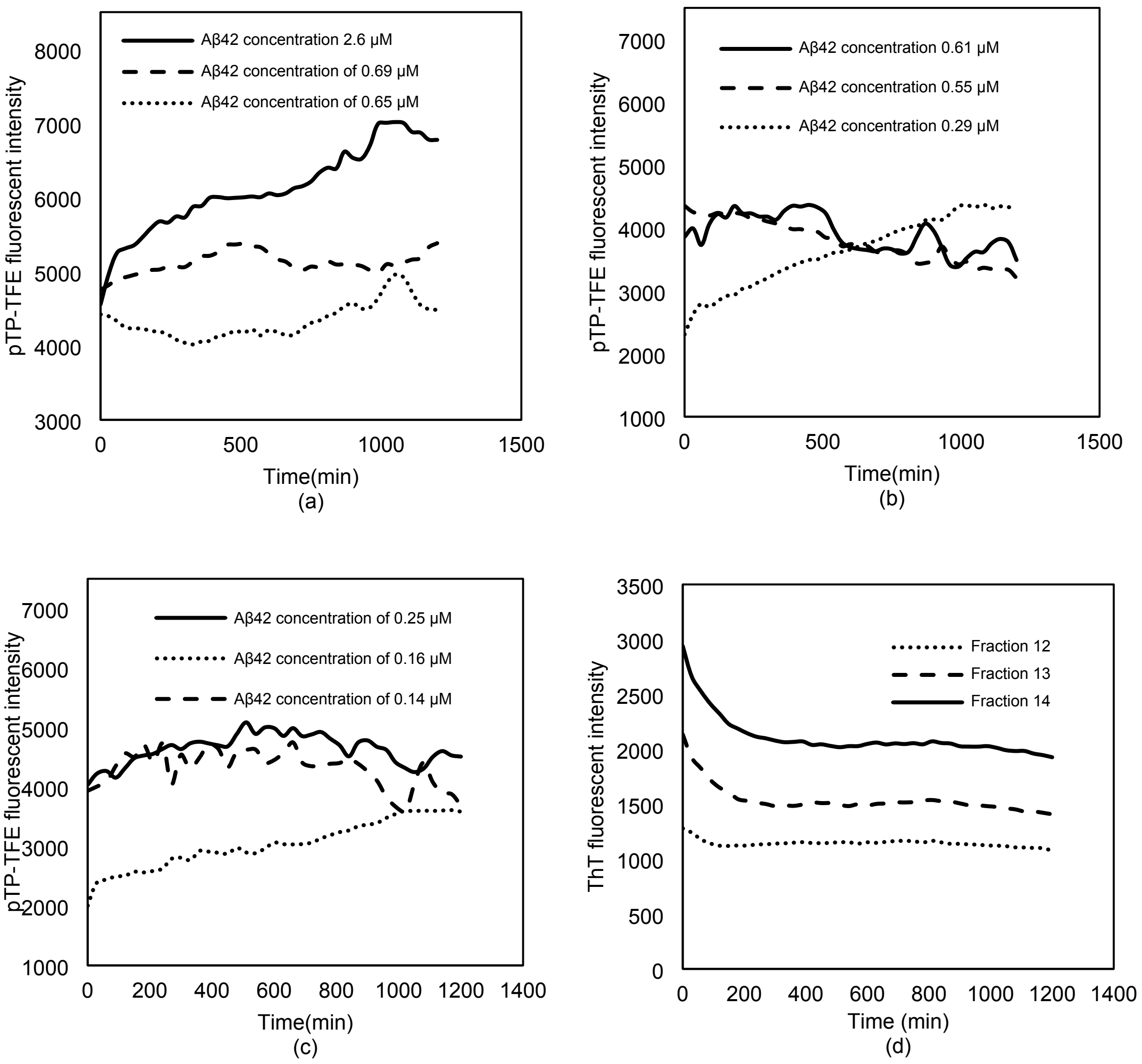

Figure S6: (a) Kinetics of Aβ42 fractions 12 with pTP-TFE; (b) Kinetics of Aβ42 fractions 13 with pTP-TFE; (c) Kinetics of Aβ42 fractions 14 with pTP-TFE; and (d) Kinetics of Aβ42 fractions 12, 13 and 14 with ThT fluorescence.

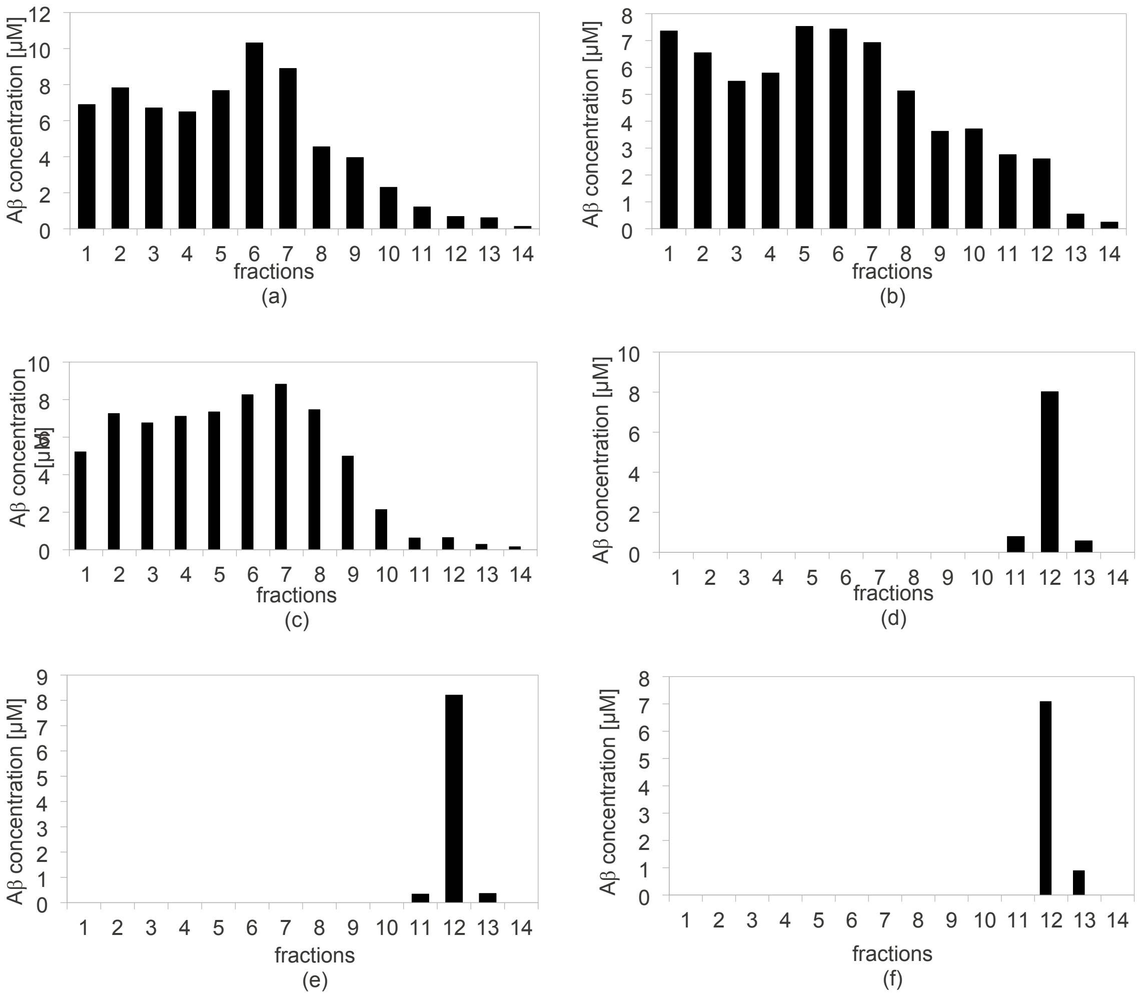

Figure S7: Aβ (1-42) concentrations. For binding affinity tests of pTP-FFE with oligomers, 140 μL of fraction 5 from each group (a)-(c) are combined. Similarly, for binding affinity tests of pTP-FFE with fibrils, 140 μL of fraction 12 from each group (d)-(f) are pooled together.
